# Maternal Mortality: An Eco-Social Phenomenon that Calls for Systemic Action

**DOI:** 10.1055/s-0040-1710041

**Published:** 2020-04

**Authors:** João Paulo Souza, Fernando Bellissimo-Rodrigues, Luciane Loures dos Santos

**Affiliations:** 1 Department of Social Medicine, Faculdade de Medicina de Ribeirão Preto, Universidade de São Paulo, São Paulo, SP, Brazil

Pregnancy, childbirth, and the postpartum period are phases commonly associated with joy and hope. Even though it may be an unplanned event for many women, pregnancy usually develops without complications most of the times: mother and newborn child start together—and well—a new phase of their lives. This does not mean that the good outcome was achieved without a significant number of women experiencing discomfort, stress, anxiety, fear, or even some sadness. These are conditions that, although not desirable, tend to be present during pregnancy, childbirth, and the postpartum period. However, for some women, this is a period of great anguish, suffering, and risk. Risk of intimate partner violence, of mistreatment in health facilities, of developing physical or psychological sequelae, and risk of dying.^1^ ^2^ ^3^ ^4^

A maternal death is an individual, family, and social tragedy. Because it is preventable in the absolute majority of times it occurs, there is no male equivalent, and it disproportionately affects certain groups of women, maternal mortality exceeds the boundaries of clinical obstetrics and reflects broader societal issues.^5^ ^6^ ^7^ While hypertensive complications, bleeding, infection, unsafe abortion, and worsening of preexisting diseases are the main biomedical causes of maternal mortality, tackling it requires broader actions.^8^ ^9^

Considered as causes of complications of pregnancy, intrinsic or extrinsic etiological agents (such as uterine atony or bacterial infection) do not act in isolation on women to produce complications. The etiological agents act under the influence of several other factors, in a complex and multifactorial process known as the health-disease process (Fig. 1). Over thousands of years, the characteristics of the environment favored the evolution of current human beings. Among the innate characteristics and potential of *Homo sapiens*, lies the biological basis of pregnancy and childbirth. This includes, for instance, the shape of the pelvis and the complex endocrinology of parturition. The innate characteristics and potential of the species favored the development, over time, of the current human culture and society. Culture and society are the origin of the guiding principles of social organization, the legal and political structure, and the mode of production of the economy. In this context, human interaction with the planet has produced environmental degradation, with consequences that include the increasing concentration of particulate matter in the atmosphere and the acceleration of global warming. The latter, besides being responsible for the melting of ice glaciers and the rise of the sea level, is associated with a greater frequency and intensity of extreme climate events, including heat waves or drought or severe storms and heavy rain. These events affect maternal health and have been associated with an increased maternal and perinatal morbidity and mortality.^7^ ^10^

**Fig. 1:**
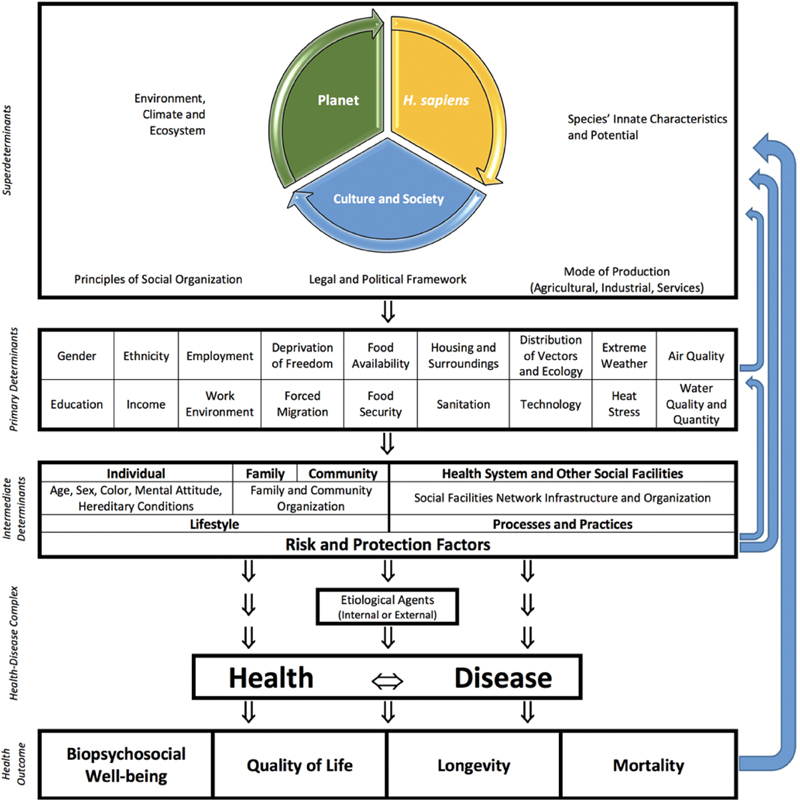
The health-disease process (eco-social model).

Together, the innate characteristics of *H. sapiens*, their culture and society, and the environment are super determinants of the whole health-disease process, thus originating the so-called primary determinants of health. Education, income, ethnicity and gender issues affect the risk of a woman dying during pregnancy, childbirth, and postpartum/postabortion period. Women of color, those living on the outskirts of large cities or in rural areas, those with little access to education or income, are the women experiencing the highest maternal mortality.^6^ Women deprived of their liberty, migrant women, women victims of trafficking and women in prostitution are frequently invisibilized and subject of additional marginalization and risk. The factors arising from or associated with the global climate emergency (e.g., extreme weather, heat stress, poor air quality, or changing distribution of infectious disease vectors) must also be highlighted.^10^ ^11^ ^12^ Under the influence of these determinants, the individual, family and community characteristics give rise to lifestyle patterns, which may accentuate or reduce risks. Likewise, the family and community organization can act as a protection and support network for women, reducing the risk of mortality, or, on the contrary, favoring harmful lifestyles. Also coming from the principles and structures of society, social facilities (such as schools and the health system itself) undertake processes and practices capable of functioning as protective factors, mitigating the negative effects of the primary determinants and enhancing their positive effects. On the other hand, by becoming permeable to structural bias, health and social facilities risk reproducing violence, including abuse, disrespect and mistreatment of women during pregnancy, childbirth, and in the postpartum/postabortion period.

Given the broad determinants of maternal mortality and the complex health-disease process, maternal mortality has long ceased to be “just” a health indicator and became a social development indicator. Hence its inclusion as a progress indicator of two successive global initiatives, the Millennium Development Goals (2000–2015) and the Sustainable Development Goals (2016–2030). Both initiatives, promoted by the United Nations (UN), seek to encourage the governments of the signatory countries to implement programs to promote social development and eliminate extreme poverty.^13^

The World Health Organization (WHO) estimates that in the early 1990s there were ∼ 500,000 maternal deaths per year worldwide. According to the UN health agency, the annual number of maternal deaths around the world would be just over 450,000 deaths in 2000 and 295,000 in 2017. The global maternal mortality ratio in 2000 and 2017 was estimated at 342 and 211 maternal deaths per 100,000 live births, respectively. In Brazil, the maternal mortality ratio in 2000 was estimated by the WHO at 69 deaths per 100,000 live births, and, in 2017, 60 deaths per 100,000 live births. The WHO estimated for 2017 a total of 1,700 maternal deaths in Brazil, with the lifetime risk of 1 maternal death for 940 women.^14^ There is some methodological difficulty in generating reliable global estimates over time, and all these estimates have a relatively wide range of unreliability. The Brazilian Ministry of Health generates its own estimates of maternal mortality. Although the estimates are compatible, considering their degree of uncertainty, the maternal mortality ratio estimated by the Brazilian Ministry of Health is slightly higher than that estimated internationally (64 maternal deaths per 100,000 live births in 2017). Within the scope of the Sustainable Development Goals, the target is to achieve a global maternal mortality ratio of 70 maternal deaths per 100,000 live births in 2030. For the global target to be achieved, each country needs to contribute to a certain reduction in mortality. For Brazil, the target maternal mortality ratio for 2030 is 30 maternal deaths per 100,000 live births.^15^

Considering the evolution of the maternal mortality ratio in Brazil since 1990, the most substantial reduction took place in the last decade of the 20th century. This reduction of maternal mortality has been partially and ecologically attributed to a greater access to primary health care during pregnancy (i.e., antenatal care), greater coordination between the different levels of the health system, and improvements in emergency services. These advances occurred in the context of greater economic stability and the implementation of the Unified Health System (SUS) in Brazil, which occurred in the beginning of the 1990s. In the 2000s, the rate of reduction in the maternal mortality ratio decreased and started to tend to stability, suggesting the need for more intense social transformations as well as greater gains in efficiency and quality in the health system.^15^ ^16^

Although there is no shortcut to reduce maternal mortality—social development is necessary for substantial and sustainable gains—the health sector cannot be exempted from its central role in tackling maternal mortality. The reduction in maternal mortality occurs over a long journey, which can be divided into stages. According to the theory of obstetric transition (Box 1), Brazil is between stages III and IV of this transition.^17^ At this point, although issues of access to health care may persist, the quality of care becomes a major determinant of pregnancy outcomes. Eliminating delays within the system itself becomes a priority. It is important to note an apparent contradiction: while maternal mortality is largely preventable, a sizable number of women will experience complications almost inevitably. The preventability of some of the main complications (for example, preeclampsia and postpartum hemorrhage) has limitations, and their prompt recognition and proper management are essential. Thus, delays in recognizing complications by women themselves or health professionals, in the decision to seek help, in obtaining access to the health system, as well as in receiving adequate, respectful and quality care in health facilities become significant determinants of maternal mortality. In this context, health and social facilities—particularly the health system—function as safeguards and protection networks: the ability to neutralize the negative effects of primary determinants can be a measure of their efficiency, whereas the system's permeability to the primary determinants can indicate the opposite. Thus, it is essential that structuring actions are implemented and developed with a goal to strengthen the health system and reduce the system response time (Box 2).^9^ ^17^ ^18^ ^19^

**Box 1:** The obstetric transition

The obstetric transition is a theory about the pathway for maternal mortality reduction. It considers the levels of maternal mortality and fertility, the pattern of biomedical causes and care. The obstetric transition is divided in stages, and at country level; it is highly related to the degree of social development. • **Stage I** (maternal mortality ratio > 1,000 maternal deaths/100,000 live births): Most women experience a situation that is close to the natural history of pregnancy and childbirth. Stage I is characterized by a very high maternal mortality rate, with high fertility and a predominance of direct causes of maternal mortality, in addition to a large proportion of deaths attributable to communicable diseases, such as malaria. Most women do not receive professional obstetric care or have access to health facilities. The priority at this stage is the promotion of social development and primary prevention measures including: reproductive planning, iron supplementation, insecticide-treated mosquito nets and the removal of barriers to access the health system. • **Stage II** (maternal mortality rate: 999–300 maternal deaths/100,000 live births): mortality and fertility remain very high, with a pattern of causes similar to that of stage I. However, a greater proportion of women begin to seek and receive care in health units. The priorities at this stage are similar to those at stage I. • **Stage III** (maternal mortality ratio: 299–50 maternal deaths/100,000 live births): fertility is variable, and the direct causes of mortality still predominate. This is a complex stage, because access remains a problem for a large part of the population. However, as a growing proportion of pregnant women reach health services, the quality of care is a major determinant of health outcomes, particularly related to overwhelmed health services. The priorities at this stage include reducing social inequalities and improving quality of care. Primary prevention, as well as secondary and tertiary prevention, is critical to improving maternal health outcomes at this stage. In other words, the quality of care and the proper management of complications are essential to reduce maternal mortality. • **Stage IV** (maternity mortality ratio < 50 maternal deaths/100,000 live births): maternal mortality is low. There is a low fertility rate. Indirect causes of maternal mortality, especially non-communicable diseases, are increasingly important. One aspect that emerges at this stage is the growing role of medicalization as a threat to quality and improvement in health outcomes. The priority at this stage is to consolidate social gains and intensify the improvement of quality and quaternary prevention (prevention of iatrogenic diseases). • **Stage V** (all preventable maternal deaths are avoided). Maternal mortality is very low, the fertility rate is low or very low and indirect obstetric causes associated with chronic-degenerative disorders are the main causes of maternal mortality. The main challenges at this stage are the consolidation of advances against structural violence, effective management of vulnerable populations (for example, immigrants, refugees and displaced persons in their own country), and sustainability of excellence in quality of care.

**Box 2:** Interventions to improve quality of care in maternity and women's health services

• Implementation of social control and community participation in maternity and women's health services, with representatives of female users of the service, health professionals, and managers (i.e., a Health Facility Council); • Implementation of a Quality Control Commission, responsible for: ○ Analyzing the infrastructure, process, and health outcomes indicators, including users' satisfaction; ○ Identifying obstacles to the provision of **women-centered, timely, appropriate and respectful quality care guided by the best scientific evidence** ○ Proposing solutions to overcome the identified obstacles • Generate actionable information through: ○ Electronic information systems ○ Audit and feedback of selected near-miss cases and all maternal and perinatal deaths. Feedback to professionals and teams is essential. • Redesign local maternal mortality committees into instances of audit and feedback or quality control commissions. The committees need to strive for local impact through feedback to local teams. • Systematize assistance through guidelines, protocols and standard operating procedures based on scientific evidence, including communication protocols and teamwork. Consider adopting structured emergency response packages (for example, ALSO, ALARM, FAST-M, surviving sepsis campaign care bundles); • Implementation of standard operating procedures, protocols, and guidelines into clinical practice through: ○ Local handbooks ○ Physical and electronic reminders ○ Local opinion leaders ○ Drills and simulations

## Conclusion

Maternal mortality is a difficult puzzle to solve. However, progress made in the last few decades is encouraging. In Brazil, the major obstacle is to advance the quest for social justice, particularly from an ethnic and gender perspective, expanding women's access to education and income, with special emphasis on women of color. In addition, it is essential to make a leap in the quality and effectiveness in public health services. The provision of women-centered, timely, appropriate and respectful quality care guided by the best scientific evidence should be the major goal of all maternity and women's health services. For this, the state needs to strengthen the public sector health system, and the society needs to exercise its primary role of defense and social control of the public health system.
